# Computed tomographic findings of macrolide-resistant *Mycobacterium massiliense* pulmonary disease and changes after antibiotic treatment

**DOI:** 10.1097/MD.0000000000016826

**Published:** 2019-09-06

**Authors:** Hyun Jung Yoon, Myung Jin Chung, Won-Jung Koh, Byung Woo Jhun, Seong Mi Moon

**Affiliations:** a Department of Radiology, Samsung Medical Center, Sungkyunkwan University School of Medicine; b Department of Radiology, Veterans Health Service Medical Center; c Division of Pulmonary and Critical Care Medicine, Department of Medicine, Samsung Medical Center, Sungkyunkwan University School of Medicine, Seoul; d Division of Pulmonary and Critical Care Medicine, Department of Medicine, Samsung Changwon Hospital, Sungkyunkwan University School of Medicine, Changwon, Korea.

**Keywords:** computed tomography, drug resistance, macrolides, *Mycobacterium massiliense*, nontuberculous mycobacteria

## Abstract

The purpose of this study was to present the computed tomographic (CT) findings of lung abnormalities in macrolide-resistant *Mycobacterium massiliense* pulmonary disease and its changes in follow-up CT after antibiotic treatment.

Chest CT scans of patients with macrolide-resistant *M massiliense* pulmonary disease (n = 19) were retrospectively reviewed. Patients were treated with multidrug therapy, and sputum examinations were performed. Follow-up CT scans obtained during antibiotic treatment after detection of macrolide resistance were also reviewed, if available (n = 13). The CT scores at detection of macrolide resistance and at the last follow-up periods were also compared.

Of all patients with macrolide-resistant *M massiliense* pulmonary disease, 2 (11%) patients achieved sputum culture conversion during the follow-up period. The most common CT findings of *M massiliense* pulmonary disease at detection of macrolide resistance were bronchiectasis and bronchiolitis (n = 19, 100%), followed by consolidation (n = 16, 84%), cavities (n = 11, 58%), and nodules (n = 6, 32%). On the last follow-up CT, overall CT scores were increased in 8 (62%) of 13 patients, and total mean CT score was significantly increased (*P* = .021). For each CT pattern, the cavity showed the greatest increase in CT score (*P* = .027), followed by bronchiectasis (*P* = .038).

Common CT findings of macrolide-resistant *M massiliense* pulmonary disease were similar to those of pulmonary disease caused by other species of nontuberculous mycobacteria at presentation. However, in macrolide-resistant *M massiliense* pulmonary disease, serial CT scans showed deterioration with cavitary and bronchiectatic change in most patients despite multidrug antibiotic therapy.

## 1 Introduction

Nontuberculous mycobacteria (NTM), a ubiquitous mycobacteria that causes chronic human pulmonary infection, has been reported to be increasing worldwide.^[1,2]^*Mycobacterium avium* complex (MAC), *Mycobacterium abscessus*, and *Mycobacterium kansasii* are the most frequent causes of NTM pulmonary disease.^[3,4]^*M abscessus* is a rapidly growing mycobacterium and the most common cause of rapidly growing mycobacterial pulmonary disease. Currently, *M abscessus* can be divided into 3 subspecies: *M abscessus* subspecies *abscessus* (hereafter referred to as *M abscessus*), *M abscessus* subspecies *massiliense* (hereafter referred to as *M massiliense*), and *M abscessus* subspecies *bolletii* (hereafter referred to as *M bolletii*).^[5,6]^ The most common subspecies is *M abscessus* (45–65%), followed by *M massiliense* (20–55%) and *M bolletii* (1–18%).^[7]^

The response rates for macrolide-based antibiotic therapy are much higher among patients with *M massiliense* pulmonary disease than for those with *M abscessus* pulmonary disease.^[8–15]^ However, acquired macrolide resistance can develop during macrolide-containing antibiotic treatment of *M massiliense* pulmonary disease and is conferred by mutations in the drug-binding receptor rrl gene for 23S rRNA, at nucleotide positions 2058 and 2059.^[16–19]^ Therefore, an awareness of macrolide-resistant *M massiliense* pulmonary disease is very important for the diagnosis and management of this disease.^[4]^

Although a recent study reported clinical characteristics of macrolide-resistant *M massiliense* pulmonary disease and the treatment outcomes of affected patients,^[20]^ there has been no report regarding the computed tomographic (CT) imaging findings of macrolide-resistant *M massiliense* pulmonary disease and changes in follow-up CTs of affected patients. One previous study presented CT findings of *M massiliense* pulmonary disease, but all *M massiliense* strains described were susceptible to macrolides.^[21]^ Thus, the purpose of our study was to demonstrate CT findings of lung abnormalities at the time of diagnosis of macrolide-resistant *M massiliense* pulmonary disease and its serial changes on follow-up CT after treatment with antibiotic therapy.

## 2 Materials and methods

This retrospective study was approved by the institutional review board of the Samsung Medical Center (IRB file No. 2018–08–001) and informed consent was waived for the use of patients’ medical data due to the retrospective nature of this study.

### 2.1 Patients and diagnoses

All patients diagnosed with macrolide-resistant *M massiliense* pulmonary disease at Samsung Medical Center between September 2005 and October 2015 were screened and their medical records reviewed. A total of 19 patients with macrolide-resistant *M massiliense* pulmonary disease for whom CT scans at the time of detection of macrolide resistance were included. The patients fulfilled the diagnostic criteria for NTM pulmonary disease according to the guidelines of the American Thoracic Society and Infectious Diseases Society of America.^[3]^ All patients were administered antibiotic therapy after macrolide-resistance detection for the disease. Fifteen patients who were described in the recently published article by Choi et al^[20]^ were included in our study. Clinical data for the 15 patients was also included in a previously published article from our institution.^[20]^

Sputum smears and cultures of acid-fast bacillus (AFB) were regularly obtained during the follow-up period.^[22]^ NTM species were identified by polymerase chain reaction (PCR)-restriction fragment length polymorphism analysis of the *rpoB* gene or reverse-blot hybridization of *rpoB*.^[8,10]^ Drug susceptibility testing was performed at the Korean Institute of Tuberculosis, using the broth microdilution method.^[23]^

### 2.2 Treatment and evaluation of treatment outcomes

For the initiation phase of treatment of macrolide-susceptible *M massiliense* pulmonary disease, patients were hospitalized for 2 or 4 weeks and received oral macrolide and/or fluoroquinolone, along with intravenous amikacin and cefoxitin (or imipenem). After discharge, the patients underwent a 2-drug oral regimen consisting of oral macrolide and/or fluoroquinolone for the continuation phase of treatment of *M massiliense* pulmonary disease in our institution.^[10]^

For the treatment of macrolide-resistant *M massiliense* pulmonary disease, a standardized treatment protocol was not established in our institution. Patients with mild symptoms when macrolide resistance was detected received oral antibiotics at the outpatient clinic. Patients with severe symptoms were hospitalized and received intravenous amikacin and cefoxitin for 2 to 4 weeks. For the oral antibiotics, treatment with a macrolide was continued for all patients and additional drugs (such as a fluoroquinolone, doxycycline, linezolid, clofazimine, or inhaled amikacin) were used which were guided by drug susceptibility results and patient tolerance.^[4,20,24]^ Sputum culture conversion after the detection of macrolide-resistant *M massiliense* pulmonary disease was assessed; conversion was defined as 3 consecutive negative cultures, with the time of conversion defined as the date of the first negative culture.^[8,10]^

### 2.3 CT acquisition

All CT examinations were performed using various helical CT scanners (Aquilion 64, Toshiba Medical System, Tokyo, Japan; LightSpeed 16, LightSpeed VCT and Discovery CT750 HD, GE Healthcare, Waukesha, WI; Brilliance-40, Philips Medical Systems, Cleveland, OH; SOMATOM Definition Flash, Siemens, Forchheim, Germany). CT scans were obtained from the lung apices to the level of the middle portion of both kidneys. All CT data were reconstructed using a high-spatial-frequency algorithm. The CT images were obtained using the following parameters: collimation, 1.25 or 0.625 mm; field of view, 36 cm; beam pitch, 1.35 or 1.375; gantry speed, 0.5 or 0.6 s/rotation; 120 kVp; 150–200 mA; and reconstruction interval, 12.5 mm. The image data were reformatted with a 2.5-mm section thickness for transverse images and a 2.0-mm section thickness for coronal images. The reconstructed images were then interfaced directly with a picture archiving and communication system (Centricity 2.0; GE Healthcare, Mt. Prospect, IL), which displayed all image data on 2 monitors (1536 × 2048 matrix, 8-bit viewable gray scale, and 60-ft-Lambert luminescence). Both mediastinal (width, 400 HU [Hounsfield unit]; level, 20 HU) and lung (width, 1500 HU; level, –700 HU) window images were viewed on these monitors.

### 2.4 CT interpretation

Two chest radiologists jointly assessed the CT images, and decisions on CT findings were reached by consensus (with 5 and 24 years of experience in chest CT interpretation, respectively). The presence of all parenchymal abnormalities in each lobe (6 lobes: right upper lobe, right middle lobe, right lower lobe, upper division of left upper lobe, lingular division of left upper lobe, and left lower lobe) was recorded. Each lung lobe was evaluated for the presence and extent of parenchymal abnormalities, including bronchiectasis, cellular bronchiolitis (small centrilobular nodules <10 mm in diameter and branching nodular structures [i.e., tree-in-bud sign]), nodules (10–30 mm in diameter), air-space consolidation (lobular [consolidation of 10–20 mm in diameter with a polygonal shape], segmental, or peribronchial), and cavities. The laterality (unilateral or bilateral) and location of lung lesions was also analyzed. A total of 114 lung lobes in 19 patients (6 lobes per patient) with macrolide-resistant *M massiliense* pulmonary disease were evaluated for the presence of lung lesions. The last follow-up CT scans were available in 13 patients, which meant the 13 patients had >1 follow-up CT scan preceded by the CT scan obtained at the time of macrolide-resistance detection, and they were also assessed in the same manner. Additionally, the time interval between the CT scan date when macrolide-resistance was detected (time point A) and the last follow-up CT scan date (time point B) was recorded.

After the pattern and distribution of the parenchymal abnormalities seen at CT were analyzed, the diseases were classified into 3 forms: fibrocavitary form (previously called the upper lobe cavitary form), nodular bronchiectatic, and unclassifiable. The fibrocavitary form was defined as when a cavity (or cavities) was present in the upper lobes with findings of emphysematous change in the middle and lower lung zones with or without a volume decrease of the upper lobes and apical pleural thickening.^[25,26]^ The nodular bronchiectatic form was defined as when bilateral bronchiectasis and cellular bronchiolitis were present mainly in the right middle lobe and lingular division of the left upper lobe, irrespective of the presence of cavities in both lungs. However, in this form, there was neither upper lobar volume loss nor emphysematous change in the remaining lungs.^[25,26]^ When the disease did not belong to either the upper lobe cavitary or the nodular bronchiectatic form, it was deemed unclassifiable. In this form, multifocal lobular or segmental consolidation or consolidation along the bronchovascular bundles might be seen.

### 2.5 CT scoring

The CT scores in terms of the severity of lung involvement in macrolide-resistant *M massiliense* pulmonary disease (Table 1) were calculated by adopting the previously published scoring system proposed by Kim et al.^[21]^ A total score of 30 was allocated for the overall extent of a lung lesion in each patient. Scores were given by considering the presence, severity, and extent of bronchiectasis, cellular bronchiolitis, cavities, nodules, and consolidation in both lungs. For cavities, the diameter, wall thickness, and extent were evaluated. The mean overall CT score for each pattern of parenchymal abnormality was defined as the sum of score of the 19 patients divided by the total number of patients. The available 13 patients’ last follow-up CT scans were also scored and recorded.

**Table 1:**
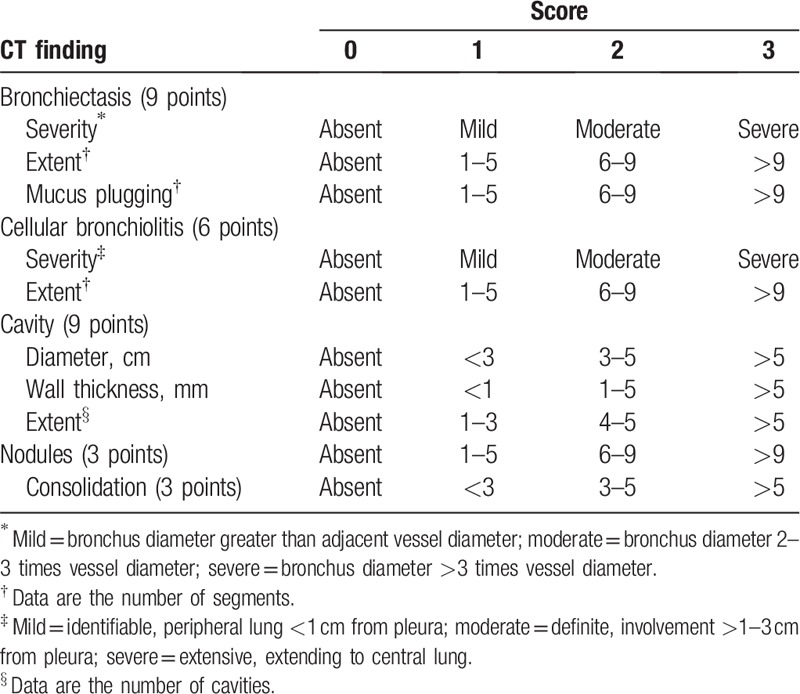
CT scoring system for assessment of the extent of macrolide-resistant *M massiliense* pulmonary disease.

### 2.6 Statistical analysis

Data are presented as the median and interquartile range (IQR) for continuous variables and as the frequency and percentage for categorical variables. CT scores of total and each parenchymal abnormality between the 2 time points were compared within the 13 patients who had follow-up CT scans as pairwise comparisons using a Wilcoxon signed rank test. A *P*-value of <.05 was considered to indicate a significant difference. Data were analyzed using IBM SPSS Statistics for Windows (version 18.0; IBM, Armonk, NY).

## 3 Results

### 3.1 Clinical characteristics and treatment outcomes

Of the 19 patients with macrolide-resistant *M massiliense* pulmonary disease, 5 patients were men and 14 patients were women, and median age was 57 years (IQR: 53–67 years). For antibiotic therapy after macrolide-resistance detection for the disease, the median period of treatment was 28 months (IQR: 12–39 months). Negative sputum conversion and its maintenance for >12 months were accomplished in only 2 patients (11%; follow-up periods after the detection of macrolide resistance were 47 and 55 months, respectively). Surgical resection was performed for 2 patients during follow-up after the detection of macrolide resistance. Therefore, the 2 patients were excluded from CT score comparison analysis. Of the 2 patients who achieved negative sputum conversion, 1 patient was who had no change in the overall CT score at the last follow-up CT and the other one was who underwent surgical resection after the detection of macrolide resistance.

### 3.2 CT Findings at time of the detection of macrolide resistance

**Figure 1:**
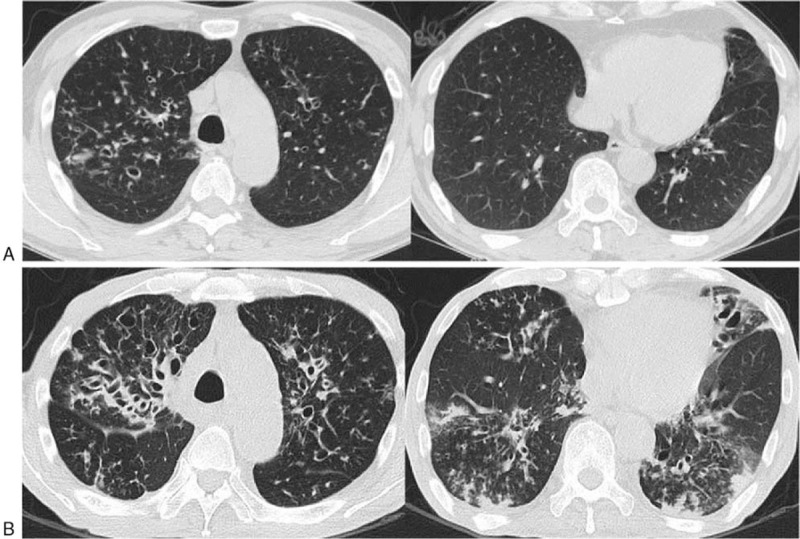
Serial CT scans of *M massiliense* pulmonary disease at time points A and B in a 66-year-old man. (A) Scans obtained at time point A show bronchiectasis and bronchiolitis mainly in both upper lobes. (B) Scans obtained at time point B (38 months after time point A) show interval progression of bronchiectasis with wall thickening of dilated bronchi (severity) in both upper lobes. The number (extent) of involved lobes was also increased. Multifocal peribronchial consolidations were increased or newly appeared in both lower lung zones. Total (severity, extent, and mucus plugging) scores for bronchiectasis, cellular bronchiolitis, and consolidation were 5, 6, and 1, respectively, for time point A and 7, 6, and 2, respectively, for time point B. CT = computed tomographic.

**Figure 2:**
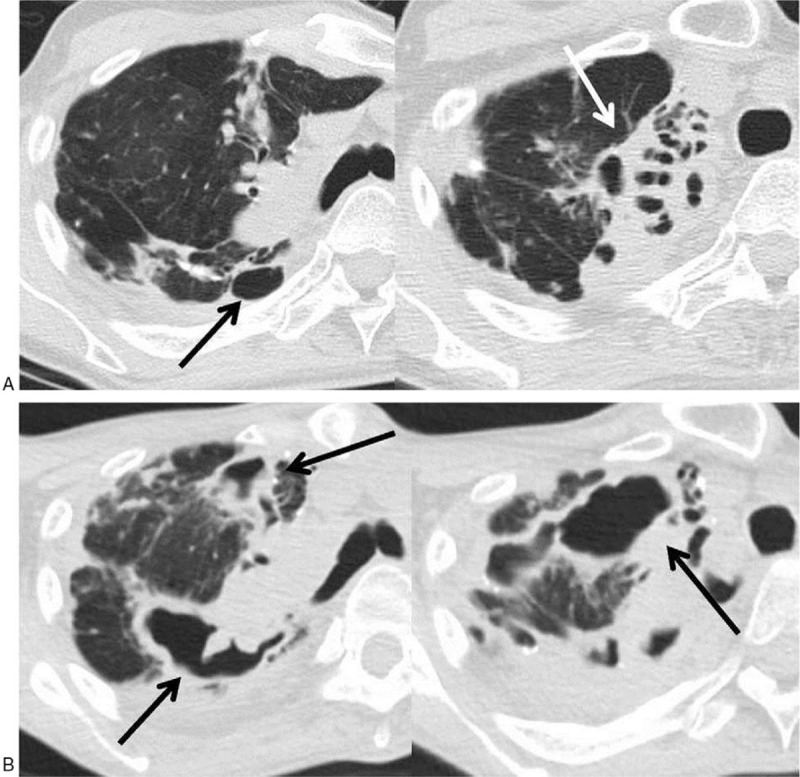
Serial CT scans of *M massiliense* pulmonary disease at time points A and B in a 46-year-old woman. (A) Scans obtained at time point A show bronchiectasis and cavities (arrows) in the right upper lung zone. (B) Scans obtained at time point B (36 months after time point A) show interval progression of cavities (arrows). Total scores (diameter, wall thickness, and extent) for cavitary lesions were 4 (score of 1, 2, and 1, respectively) for time point A and 7 (scores of 3, 3, and 1, respectively) for time point B. CT = computed tomographic.

Of 19 patients, 10 (53%) patients had the nodular bronchiectatic form, 7 (37%) had the fibrocavitary form, and 2 (10%) had the unclassifiable form. The pattern of the parenchymal findings including the frequency, laterality, and location of the lung lesions are summarized in Table 2. The most common CT findings at time of the detection of macrolide resistance were bronchiectasis and bronchiolitis (n = 19, 100%) (Fig. 1), followed by consolidation (n = 16, 84%), cavities (n = 11, 58%; Fig. 2), and nodules (n = 6, 32%). Cellular bronchiolitis and bronchiectasis were bilateral in distribution in 89% of patients, and they involved more than two-thirds of all lung lobes.

**Table 2:**
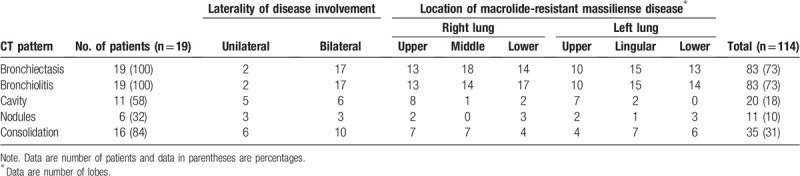
Pattern and distribution of parenchymal abnormalities on CT at the time of diagnosis of macrolide-resistant *M massiliense* pulmonary disease.

The CT scores recorded by both observers are shown in Table 3. In all 19 patients of macrolide-resistant *M massiliense* pulmonary disease, bronchiectasis and cellular bronchiolitis has relatively higher scores (4.9 and 4.9, respectively) than those of other disease patterns.

**Table 3:**
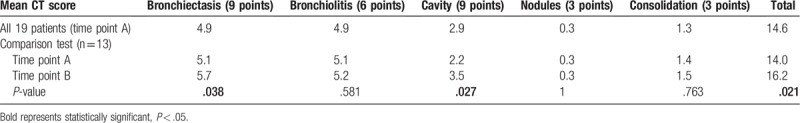
Mean CT scores in terms of disease patterns and comparison of scores.

### 3.3 Changes in the last CT findings after treatment

In comparing CT scores between the time point A and B in the 13 patients who had follow-up CT scans, 8 (62%) of 13 patients’ scores increased, 2 (15%) decreased, and 3 (23%) had no change in the overall CT score at the last follow-up CT (time point B). The total mean CT score was significantly increased at time point B (*P* = .021). In CT patterns, cavities showed the greatest increase in score (*P* = .027; Fig. 1), followed by bronchiectasis (*P* = .038; Fig. 2). Cellular bronchiolitis and consolidation showed a slight increase in score, but these increases were not statistically significant (*P* = .581 and .763, respectively). Nodules showed no change in score. The median time interval between the time points A and B was 24 months (IQR: 10–37 months).

## 4 Discussion

There have been several studies regarding CT imaging findings of NTM pulmonary diseases caused by *M abscessus* and *M massiliense* or MAC. Considerable overlap exists in the imaging findings among those diseases.^[21,25,27,28]^ Kim et al^[21]^ demonstrated the predominant CT findings of macrolide-susceptible *M massiliense* to be bilateral bronchiectasis and cellular bronchiolitis or upper lobe cavities combined with consolidations. However, there has been no previously published report regarding the CT imaging findings of macrolide-resistant *M massiliense* pulmonary disease in detail.

In this study, we investigated CT imaging findings of 19 patients with macrolide-resistant *M massiliense* pulmonary disease as well as its final changes in comparable 13 patients upon follow-up. Similar to the above-mentioned Kim et al study,^[21]^ the most common CT findings at presentation in macrolide-resistant *M massiliense* pulmonary disease in our study were bilateral cellular bronchiolitis and bronchiectasis (n = 19, 100%). Cavities were noted in 11 (58%) patients. Among the 19 patients, the nodular bronchiectatic form is more frequent than the fibrocavitary form; this result was also similar to the previous study by Kim et al.^[21]^ Ten (53%) patients had the nodular bronchiectatic form, 7 (37%) had the fibrocavitary form, and 2 (10%) had the unclassifiable form in our study. However, in comparison to the previous report, macrolide-resistant *M massiliense* pulmonary disease had a greater tendency to include cavities than macrolide-susceptible *M massiliense* lung disease (11 [58%] vs 45 [44%]), and to be the fibrocavitary form (7 [37%] vs 8 [24%]).^[21]^ From these comparisons, we could assume that CT findings of *M massiliense* at the time of detection of macrolide-resistance have a tendency to present cavitary change, because macrolide resistance could develop after long-term antibiotic therapy which included macrolide in patients with *M massiliense* pulmonary disease.

*M abscessus* pulmonary disease has been shown to have unsatisfactory clinical and radiographic treatment success rates (25–42%; 8, 28). In contrast, *M massiliense* pulmonary disease reported high negative sputum conversion rates and radiographic improvement rates after antibiotic therapy^[8,10,21,29]^; this may be because *M abscessus* has inducible macrolide resistance, but inducible resistance is not found in *M massiliense*, which has a partially deleted, nonfunctional *erm*(41) gene.^[16]^ However, once macrolide-resistant is detected, the expected course of disease changes dramatically. Choi et al^[20]^ reported that the treatment outcomes of macrolide-resistant *M massiliense* pulmonary disease were very poor after multidrug antibiotic treatment. In the report, only one (7%) of 15 patients had a favorable outcome, and the 5-year mortality rate after the development of macrolide resistance was high (33%). Another previous report suggested that susceptibility to macrolide was the only significant independent predictor of a favorable microbiological response in *M abscessus* and *M massiliense* pulmonary disease.^[30,31]^ Our study also showed low negative sputum conversion rate (11%).

In our study, the total mean CT score was significantly increased at final follow-up CT scans (*P* = .021) despite long-term antibiotic treatment, and cavities showed the greatest increase in mean score (*P* = .027), followed by bronchiectasis (*P* = .038). This implies that the phenotype of macrolide-resistance *M massiliense* pulmonary disease gradually increases in irreversible cavities and bronchiectasis, eventually leading to deterioration. In other words, even though nodular bronchiectatic form is radiologically presented on initial CT in *M massiliense* pulmonary disease, once macrolide-resistance detected, it may finally present fibrocavitary form or profuse cavities and bronchiectasis on imaging which imply end stage of *M massiliense* pulmonary disease. Despite the emergence of resistance, limited data for the imaging findings on macrolide-resistant *M massiliense* pulmonary disease are available and only clinical aspects were reported on previous work.^[20]^ Thus, our report is meaningful in terms of the first presenting report regarding radiologic findings of macrolide-resistant *M massiliense* pulmonary disease and building baseline research data for the next relevant study. In 2 patients, CT scores were decreased in the last CT scan; time intervals between the time points A and B were 5 and 132 months. CT findings of these patients were classified as nodular bronchiectatic form, and bronchiolitis and consolidation (readily reversible pattern) were the main pattern. Neither of these patients had cavities. From these results, we infer that the dominant CT pattern may predict treatment outcomes in macrolide-resistant *M massiliense* pulmonary disease. Bronchiectasis and cavitations are permanent lung changes to infection and their morphology should not be expected to change to any significant extent in response to effective treatment. In contrary, bronchiolitis and consolidation are more acute changes of lung infection with potential for complete reversibility on effective treatment. However, our data showed morphologic imaging alone and it may not be effective for therapy response assessment in patients with permanent lung change. Several previous reports demonstrated usefulness of metabolic imaging such as F-18 FDG PET/CT in the assessment of treatment response of mycobacterial disease and time course of the disease. Thus, F-18 FDG PET/CT imaging may have the possible role of a potential imaging tool for response assessment also in macrolide-resistant *M massiliense* pulmonary disease.^[32,33]^ Further research is needed in conjunction with establishing optimal treatment regimens for *M massiliense* pulmonary disease, especially macrolide-resistant disease.

There were several limitations in our study. First, it was conducted at a single referral center and the number of included patients was too small to achieve strong statistical power. Second, our study was retrospective in design; thus, it may have had selection bias. We included only patients who had the CT at the time of detection of macrolide-resistance and follow-up CT scans. Also, we excluded 2 patients in the comparative analysis because they had surgical treatment for localized cavitary lesions after detection of macrolide-resistance. Third, the time point B was variable, (IQR: 10–37 months in comparison analysis); however, this might not affect the overall results because the median time interval was long enough to show radiological change on follow-up CT scan. Fourth, the CT scoring by 2 radiologists was combined using a consensus. Consequently, the senior radiologist will likely be more influential in consensus statements.

## 5 Conclusion

The most common CT findings at presentation in patients with macrolide-resistant *M massiliense* pulmonary disease are cellular bronchiolitis, bronchiectasis, consolidation, cavitary lesions, and nodules in a decreasing order of frequency. The nodular bronchiectatic form is more common than the fibrocavitary form. Most patients with macrolide-resistant *M massiliense* pulmonary disease showed deterioration in their CT findings, with gradual changes in cavities and bronchiectasis during antibiotic treatment. Because patients with macrolide-resistant *M massiliense* pulmonary disease show a poor response to antibiotic therapy in the sputum and on imaging studies, accurate and timely detection of macrolide-resistance in *M massiliense* pulmonary disease during treatment is necessary.

## Author contributions

**Conceptualization:** Hyun Jung Yoon, Myung Jin Chung, Won-Jung Koh.

**Data curation:** Hyun Jung Yoon, Myung Jin Chung, Won-Jung Koh, Byung Woo Jhun, Seong Mi Moon.

**Formal analysis:** Hyun Jung Yoon, Myung Jin Chung, Byung Woo Jhun, Seong Mi Moon.

**Funding acquisition:** Won-Jung Koh.

**Investigation:** Hyun Jung Yoon, Won-Jung Koh.

**Methodology:** Hyun Jung Yoon, Myung Jin Chung, Won-Jung Koh, Byung Woo Jhun, Seong Mi Moon.

**Supervision:** Myung Jin Chung, Won-Jung Koh.

**Writing – original draft:** Hyun Jung Yoon.

**Writing – review & editing:** Myung Jin Chung, Won-Jung Koh, Byung Woo Jhun, Seong Mi Moon.

Myung Jin Chung orcid: 0000-0002-6271-3343.
